# Revision surgery of metal-on-metal hip arthroplasties for adverse reactions to metal debris: A clinical update

**DOI:** 10.1080/17453674.2018.1440455

**Published:** 2018-03-01

**Authors:** Gulraj S Matharu, Antti Eskelinen, Andrew Judge, Hemant G Pandit, David W Murray

**Affiliations:** 1 Nuffield Department of Orthopaedics, Rheumatology and Musculoskeletal Sciences, University of Oxford, Nuffield Orthopaedic Centre, Oxford, United Kingdom; 2 Coxa Hospital for Joint Replacement, Tampere, Finland

## Abstract

**Background and purpose:**

The initial outcomes following metal-on-metal hip arthroplasty (MoMHA) revision surgery performed for adverse reactions to metal debris (ARMD) were poor. Furthermore, robust thresholds for performing ARMD revision are lacking. This article is the second of 2. The first article considered the various investigative modalities used during MoMHA patient surveillance (Matharu et al. 2018a). The present article aims to provide a clinical update regarding ARMD revision surgery in MoMHA patients (hip resurfacing and large-diameter MoM total hip arthroplasty), with specific focus on the threshold for performing ARMD revision, the surgical strategy, and the outcomes following revision.

**Results and interpretation:**

The outcomes following ARMD revision surgery appear to have improved with time for several reasons, among them the introduction of regular patient surveillance and lowering of the threshold for performing revision. Furthermore, registry data suggest that outcomes following ARMD revision are influenced by modifiable factors (type of revision procedure and bearing surface implanted), meaning surgeons could potentially reduce failure rates. However, additional large multi-center studies are needed to develop robust thresholds for performing ARMD revision surgery, which will guide surgeons’ treatment of MoMHA patients. The long-term systemic effects of metal ion exposure in patients with these implants must also be investigated, which will help establish whether there are any systemic reasons to recommend revision of MoMHAs

Many metal-on-metal hip arthroplasties (MoMHAs) have been implanted worldwide in the form of hip resurfacing arthroplasty (HRA) and total hip arthroplasty (THA) (Bozic et al. 2009, NJR 2016). Despite the high failure rates of MoMHAs (Smith et al. 2012a, 2012c), it is estimated that at least 80% of these implants remain in situ worldwide (AOANJRR 2016, NJR 2016). Adverse reactions to metal debris (ARMD) represent an almost unique mode of arthroplasty failure associated with MoMHAs. ARMD is very different from conventional modes of arthroplasty failure, such as dislocation, loosening, and infection, given the associated soft-tissue problems, the potentially progressive and destructive disease nature, as well as its development in patients with asymptomatic and seemingly well-functioning MoMHAs (Grammatopoulos et al. 2009, Liddle et al. 2013, Matharu et al. 2016c).

Given the prevalence of ARMD revision surgery is increasing (Liddle et al. 2013, AOANJRR 2016, Matharu et al. 2016b, NJR 2016) it is expected that many more MoMHA patients will undergo future revision. However a systematic review suggested that there was little good quality evidence available regarding the outcomes following MoMHA revision surgery performed for ARMD (Matharu et al. 2014a), which represents the commonest cause of revision (Matharu et al. 2016b). This therefore makes it difficult for surgeons to counsel patients about the risks of undergoing further procedures.

Furthermore, robust thresholds for performing revision for ARMD in MoMHA patients have not been established due to a lack of evidence (Matharu et al. 2015). This is reflected in the variable recommendations proposed by worldwide regulatory authorities for considering MoMHA revision surgery (Canada 2012, MHRA 2012, Therapeutic Goods Administration 2012, FDA 2013, Hannemann et al. 2013). Knowledge of any prognostic factors of outcome following ARMD revision would assist surgeons when making decisions about the threshold (when to recommend revision) and type of revision surgery (which components require revision, and which bearing surface/fixation methods to use) required in MoMHA patients with ARMD.

This article is the second of 2, which aims to provide a clinical update on the investigation and management of MoMHA patients. The first article considered the various investigative modalities used during MoMHA surveillance, with specific focus on blood metal ion sampling and imaging (Matharu et al. 2018a). The present article considers the threshold for performing revision, the surgical strategy, and the outcomes following ARMD revision surgery when performed in patients with MoM HRAs and MoM THAs with femoral head sizes of 36 mm or greater.

## Threshold for ARMD revision surgery

The poor outcomes initially reported following ARMD revision surgery (Grammatopoulos et al. 2009, de Steiger et al. 2010) led regulatory authorities and surgeons to widely recommend performing early revision in MoMHAs with ARMD (Grammatopoulos et al. 2009, De Smet et al. 2011, Haddad et al. 2011, MHRA 2012, FDA 2013). However, there are currently no robust thresholds for performing ARMD revision surgery, because of lack of evidence. Therefore surgeons have difficulty when managing patients with ARMD: which patients to revise and which to keep under surveillance?

The limited evidence base and problems clinicians experience is highlighted by expert opinion (level 5 evidence) (Oxford 2011) commonly being used to manage these patients (Hannemann et al. 2013, Berber et al. 2015). In 2012, one UK center introduced an internet-based multidisciplinary team to assist other centers in managing their MoMHA patients with cases referred electronically (Berber et al. 2015). The multidisciplinary team consists of experts (specialist surgeons and radiologists), who meet weekly to recommend management decisions based on their experience, regulatory guidance (MHRA 2012, FDA 2013), and the latest evidence. The proposed management recommendations have been implemented by referring institutions in 92% of cases (Berber et al. 2015).

For some cases management is clear. Timely revision surgery is needed for symptomatic patients with pseudotumors that are solid, large, invasive, and destructive to the soft tissues and bone, sometimes with neurovascular damage (Pandit et al. 2008, Grammatopoulos et al. 2009, Langton et al. 2010, Langton et al. 2013, Liddle et al. 2013). Similarly, asymptomatic patients with normal investigations (no imaging abnormalities and blood metal ion levels below 2 µg/L) are also straightforward and do not require revision. This leaves a lot of patients where management remains uncertain. Such patients include those with moderate to no symptoms with abnormal investigations, which can include non-destructive cystic pseudotumors and/or moderately raised blood metal ion concentrations (Almousa et al. 2013, Hasegawa et al. 2014, Goldstein et al. 2016, Kwon et al. 2016, Matharu et al. 2016c). This uncertainty is highlighted by a recent study where 10 MoMHA clinical scenarios were used to assess the management decisions made by international experts from 6 centers (Berber et al. 2016). Agreement was inconsistent between centers specifically when managing patients with raised or rising blood metal ion concentrations, cystic pseudotumors, and peri-acetabular osteolysis (Berber et al. 2016).

The issue of systemic disease in MoMHA patients continues to generate interest. The long-term effects of high concentrations of cobalt and chromium in the body remain unknown. Patients with ARMD may develop systemic symptoms due to exposure to high cobalt and chromium concentrations. A review reported that MoMHA patients with systemic features had median serum cobalt concentrations of 35 (14–288) µg/L, with symptoms often resolving after revision to a non-MoM articulation (Zywiel et al. 2016). Systemic features appear to be extremely rare but can be divided into neurological (hearing and visual impairment/loss, peripheral neuropathy, and cognitive impairment), cardiovascular (cardiomyopathy, breathlessness), and endocrine (hypothyroidism, fatigue, malaise, depression) (Tower 2010, Bradberry et al. 2014, Cheung et al. 2016, Zywiel et al. 2016).

Deaths due to cardiac failure secondary to cobalt toxicity have been reported in MoMHA patients, even following revision (Gilbert et al. 2013, Martin et al. 2015). Analysis of Australian Veterans data reported an association between ASR XL MoM THAs and hospital admission for heart failure in elderly male patients (Gillam et al. 2017). However, a larger study using National Joint Registry (NJR) data from England and Wales demonstrated that MoMHA patients were not at increased risk of heart failure compared with non-MoMHA patients (Sabah et al. 2018), with the same authors reporting that MoMHA patients with high blood metal ion concentrations undergoing comprehensive cardiac investigations had no detectable heart pathology (Berber et al. 2017). Although an established relationship exists between high metal ion exposure and the development of certain cancers in the occupational setting (Keegan et al. 2007), population data from the NJR and Finnish Arthroplasty Register have yet to demonstrate any increased risk of cancer or mortality in MoMHA patients compared with conventional THA patients at short-term follow-up (Makela et al. 2012, McMinn et al. 2012, Smith et al. 2012b, Kendal et al. 2013, Makela et al. 2014). Currently regulatory authorities do not make recommendations for considering revision surgery in MoMHA patients presenting with systemic symptoms (Matharu et al. 2015).

## Intraoperative findings and surgical strategy

The surgical management of ARMD has evolved over time, with the heterogeneity of ARMD not being appreciated initially (Grammatopoulos et al. 2009, De Smet et al. 2011, Liddle et al. 2013). Therefore MoMHA revisions performed for ARMD can range from simple to more complex cases (Liddle et al. 2013).

Simple cases to manage surgically include synovitis with minimal tissue damage and metallosis, with a satisfactory reconstruction usually achieved with primary conventional THA implants. Furthermore, when revising MoM THAs some surgeons have advised retaining acetabular and/or femoral components when these are well fixed and well positioned, with taper adapters recommended if femoral tapers are not severely damaged (Munro et al. 2014, Lainiala et al. 2015, Plummer et al. 2016). In such instances procedures may be performed where either 1 MoMHA component is revised (acetabular or femoral), or both components are retained with exchange of the modular components only (femoral head and acetabular liner, with or without the use of a taper adapter).

Complex cases include large pseudotumors that are destructive to the peri-prosthetic bone and soft tissues, which pass through tissue planes and also involve vital neurovascular structures (such as the femoral vessels or the sciatic nerve). Such complex cases are likely to require extensive reconstruction with revision THA implants with or without augments. Occasionally staged revision procedures for ARMD may be considered for severe cases or when infection is also a concern. In addition, surgical expertise from other specialties (plastics, vascular, orthopedic oncology, or pelvic surgeons) may also be required depending on the lesion anatomy, nature of the destruction, and the neurovascular involvement. This highlights the importance of performing detailed preoperative investigations, and planning for surgeons with other expertise to be available at revision as well as the necessary reconstructive implants (Liddle et al. 2013).

Dealing with large soft-tissue lesions around MoMHAs represents a new challenge for arthroplasty surgeons. Given the potential risk of ARMD recurrence (Grammatopoulos et al. 2009, Matharu et al. 2014a), the aim of revision surgery is to excise all metal debris within the soft tissues, including pseudotumors, similar to an oncological resection. However, in reality this aim can become compromised depending on the lesion anatomy, namely if it involves vital neurovascular structures and/or the primary soft tissues contributing to hip stability.

There is little guidance available regarding the specific type of reconstruction to manage ARMD revisions in MoMHAs, namely which components to revise, and which component fixation methods and bearing surfaces to implant. On the basis of the limited current available evidence we have made some recommendations about these particular aspects given the authors have significant experience with revision of MoMHAs for ARMD (Matharu et al. 2014b, Lainiala et al. 2015, Matharu et al. 2016b).

Selective component revisions are simpler surgically compared with revising the entire construct, especially in MoM THAs. Theoretically single component and modular component only revisions reduce the risks associated with removing the acetabular and/or femoral components. However even well-fixed components, especially the acetabulum, should be removed if they are clearly malpositioned (De Smet et al. 2011). On the femoral side special attention should be paid to assess the trunnion in MoM THAs; if it is severely worn the use of a taper adapter is not appropriate. Instead the stem should be removed, with extended trochanteric osteotomies sometimes required to facilitate the removal of a well-fixed femoral component. Therefore the use of single component or modular component only revisions should not be overused, with recent NJR data reporting that employing the latter strategy when revising MoM THAs with ARMD was associated with twice the risk of re-revision compared with all component revisions and compared with hips undergoing acetabular component only revisions (Matharu et al. 2017a). Instability and infection were the commonest reasons for re-revision following these modular component only exchanges (Matharu et al. 2017a).

No studies have yet shown the superiority of one type of fixation method over another following ARMD revision. Uncemented implants have been favored at ARMD revision, presumably because patients who received MoMHAs were young and active (De Smet et al. 2011, Matharu et al. 2017a). If concerns exist about osseointegration of the revision prosthesis on the femoral side, which may be due to associated bone loss or elderly patients with osteoporotic bone, we suggest using cemented primary implants or revision implants (often uncemented), with proximal femoral replacements sometimes needed in severe cases. Uncemented revision implants with or without augments, pelvic plating, and/or cages are often used for managing bone loss on the acetabular side (Liddle et al. 2013, Munro et al. 2014, Lainiala et al. 2015). Recent NJR data in MoMHAs revised for ARMD suggested acetabular bone grafting was associated with twice the risk of re-revision compared with not grafting, with the authors suggesting that the higher failures associated with bone grafting may be either due to the need for more complex reconstructions thus necessitating the graft, or because of problems with the graft itself (such as infection) (Matharu et al. 2017a).

It is universally accepted that ARMD revisions should receive a non-MoM bearing surface, given the potential for high wear from MoM bearings (Kwon et al. 2010, Langton et al. 2010) and the poor outcomes subsequently reported when MoM bearings were used at revision (Liddle et al. 2013, Matharu et al. 2014b, Pritchett 2014). Recent NJR data observed that, in ARMD revisions, implanting ceramic-on-ceramic bearing surfaces was associated with an 86% increased risk of re-revision compared with ceramic-on-polyethylene bearings; the outcomes for metal-on-polyethylene were not significantly different when compared with either of these ceramic bearing couples (Matharu et al. 2017a). However, this study was not able to perform further analyses on the type of polyethylene (standard vs. highly cross-linked) as this particular information was not available for the authors to assess.

In cases with well-functioning abductors and good soft-tissue balance, we currently recommend using ceramic femoral heads combined with highly cross-linked polyethylene liners. This strategy also minimizes the risk of further metal wear debris and/or corrosion that can be generated when using metal-on-polyethylene revision bearings, which can be a source of ARMD recurrence (Munro et al. 2014, Whitehouse et al. 2015, Plummer et al. 2016). When stability is compromised because of soft-tissue problems, options include large-head ceramic-on-ceramic bearing surfaces, large ceramic heads with thin cross-linked polyethylene liners, dual mobility cups, or constrained acetabular liners (De Smet et al. 2011, Liddle et al. 2013, Pritchett 2014, Lainiala et al. 2015). In these complex cases with poor soft tissues there is no compelling evidence to recommend any of these options over the others (Jones 2016). However, retaining the original 1-piece MoM acetabular component (with a worn internal surface) and combining it with a Dual Mobility head could potentially increase the risk of polyethylene wear.

## Outcomes following ARMD revision surgery

### Evidence available and patient demographics

A systematic review undertaken in 2013 identified 6 studies reporting the outcomes (complications, re-revision surgery, and post-revision functional outcomes) following 216 MoMHA revisions performed for ARMD (Matharu et al. 2014a). This review was updated to include eligible studies up to the end of 2016 using the methods described (see Supplementary data). Including the 6 initial studies a total of 15 unique studies were eligible for inclusion with 803 MoMHA revisions for ARMD (Grammatopoulos et al. 2009, De Smet et al. 2011, Ebreo et al. 2011, Rajpura et al. 2011, Liddle et al. 2013, Su and Su 2013, Matharu et al. 2014b, Munro et al. 2014, Norris et al. 2014, Pritchett 2014, Cip et al. 2015, Lainiala et al. 2015, Stryker et al. 2015, van Lingen et al. 2015, Liow et al. 2016). 1 further study reported the outcomes following 16 ARMD revisions at extended follow-up (median of 10 years) (Matharu et al. 2017b). All included studies were either case-control or cohort studies graded as level 4 evidence (Oxford 2011), with the majority being retrospective. We acknowledge that a number of registry reports of outcomes following MoMHA revision surgery exist (de Steiger et al. 2010, Wong et al. 2015, Penrose et al. 2016). However, these studies were not considered formally, as they did not stratify outcomes following MoMHA revision according to the reason for revision of the primary MoMHA implant.

Included studies had certain limitations such as small sample size, and data quality problems including missing data, which can all make synthesis of the available evidence problematic. 10 of the 15 unique studies involved small cohorts of less than 50 ARMD revisions. Apart from the extended follow-up report (Matharu et al. 2017b) all studies had a mean follow-up after ARMD revision of 5 years or shorter, with most having a mean follow-up of 3 years or less. A number of studies did not specifically provide patient demographics and outcomes for the ARMD revision procedures. Some studies reported on complications and re-revision surgery but not on post-revision functional outcomes, or vice versa (Norris et al. 2014, Stryker et al. 2015).

The patient demographics and outcomes following ARMD revision surgery for all included studies are summarized in Table 1. Most revisions were performed in females, in young patients (under 60 years), and within 5 years of primary MoMHA. Revisions were almost equally split between HRAs and MoM THAs. The initial systematic review (Matharu et al. 2014a) concluded that there was limited evidence on outcomes following MoM THA revisions performed for ARMD (Munro et al. 2014). Therefore a number of studies have subsequently reported on the outcomes following MoM THA revisions.

**Table 1.** Studies reporting the outcomes following metal-on-metal hip arthroplasty revision surgery performed for ARMD

A	B	C	D	E	F	G	H	I	J
Grammatopoulos et al. (2009)	16 R	100% (16)	51.3 (20–71)	1.6^a^ (0.01–6.7)	3.0^a^ (0.8–7.2)	50% (8)	38% (6)	Dislocation ± ARMD recurrence (4) Loose cup (2)	Mean OHS 20.9
Rajpura et al. (2011)	11 R	36% (4)	53.5 (22–67)	3.8 (1.3–7.3)	1.8 (1.0–3.3)	18% (2)	18% (2)	ARMD recurrence (2)	Mean OHS 35.3
De Smet et al. (2011)	48 R	61% (NS)^a^	52.5 (18–71)	2.7 (0.3–8.4)	3.3^a^ (0.3–10.1)	≤ 23% (11)^a^	≤ 13% (6) *	Loose cup or stem (2) Infection (2) ARMD recurrence (1)	Mean HHS 93.1^a^
Ebreo et al. (2011)	42 R + T	55% (23)	Median 61 (NS)	4.7^a^ (1.3–7.8)	2.2^a^ (1.2–4.0)	≤ 10% (4)^a^	≤ 2% (1)^a^	Infection (1)	Mean OHS 23.7^a^
Liddle et al. (2013)	32 R	81% (26)	57.7 (25–74)	4.3 (0.9–10.9)	Median 2.5 (1.0–4.5)^a^	≤ 6%	≤ 6%	Dislocation (1) ARMD recurrence with loose cup (1)	Median OHS 36.5^a^
Su and Su (2013)	13 R	85% (11)	NS	NS	2.3^a^ (0.7–6.7)	≤ 15% (2)^a^	≤ 15% (2)^a^	Infection (2)	Mean HHS 96.4
Munro et al. (2014)	19 T	37% (NS)^a^	57.5 (46–76)	2.8^a^ (0.6–4.9)	2.1^a^ (0.8–4.0)	68% (13)	21% (4)	Dislocation and/or loose cup (3) ARMD recurrence (1)	Mean WOMAC (pain) 78 (function) 83
Pritchett (2014)	90 R	48% (43)	49.8 (32–71)	2.8 (1.3–4.9)	5.1 (3.0–9.8)	4% (4)	3% (3)	ARMD recurrence (1) Infection (1) Loose cup (1)	Mean HHS 93.2
Matharu et al. (2014b)	46 R 18 T	72% (46)	57.8 (31–79)	5.5 (1.1–13.8)	4.5 (1.0–14.6)	20% (13)	13% (8)	Dislocation (2) ARMD recurrence (2)	Median OHS 39
Norris et al. (2014)	35 R	71% (25)	58.0 (30–76)	4.3 (1.5–9.6)	NS	NS	NS	NS	Mean OHS 33
Cip et al. (2015)	20 T	47% (NS)^a^	49.6^a^ (21–61)	4.6^a^ (2.7–6.7)	2.3 (1.5–3.1)	10% (2)	5% (1)	Infection (1)	Mean HHS 85.1
Stryker et al. (2015)	58 T	65% (NS)^a^	60.0^a^ (17–84)	3.9^a^ (0.1–9.5)	1.2^a^ (0–10.2)	20% (23)	16% (18)	Infection (7) Loose cup or stem (6) Dislocation (4)	NS
Lainiala et al. (2015)	49 R 166 T	60% (130)	62.1 (SD 10.1)	4.7 (SD 1.3)	2.3 (1.0–NS)	5% (11)	3% (6)	Dislocation (4) Infection (1)	Median OHS 40
van Lingen et al. (2015)	38 T	69% (NS)^a^	63.0^a^ (44–75)	Median 3.7 (1.0–6.5)^a^	3.1^a^ (2.1–4.7)	24% (9)	8% (3)	Dislocation (3)	Mean HOOS 61.9^a^
Liow et al. (2016)	25 R 77 T	36% (35)	62.0 (41–85)	5.1 (1.4–18.3)	2.5 (2.2–4.3)	14% (14)	7% (7)	ARMD recurrence (3) Dislocation (2) Loose cup (2)	Mean HSS 75.6
Matharu et al. (2017b)^b^	16 R	100% (16)	51.3 (20–71)	1.6^a^ (0.01–6.7)	Median 10.3 (7–15)^a^	69% (11)	44% (7)	Dislocation ± ARMD recurrence (5) Loose cup (2)	Median OHS 21

NS = not stated, SD = standard deviation

A. Study author and year

B. Hips revised for ARMD (adverse reactions to metal debris) R: Resurfacing arthroplasty T: Total hip arthroplasty

C. Female hips, % (n)

D. Mean age (range) at revision in years

E. Mean time to revision (range) in years

F. Mean follow-up time after revision (range) in years

G. Frequency of complications, % (n)

H. Frequency of re-revision, % (n)

I. Main reasons for re-revision surgery, % (n)

j. Functional outcome: Functional outcome scoring systems: OHS (Oxford Hip Score) = 0–48 (48 best outcome) (Dawson et al. 1996, Murray et al. 2007); HHS (Harris Hip Score) = 0–100 (100 best outcome) (Harris 1969); HOOS (Hip disability and osteoarthritis outcome score) = 0–100 (100 best outcome) (Klassbo et al. 2003); WOMAC (Western Ontario and McMaster Universities Arthritis Index) = 0–100 (0 best out-come) (Bellamy et al. 1988).

a Studies did not provide the relevant data specifically for the cohort of patients undergoing revision for adverse reactions to metal debris (but rather for the whole cohort of metal-on-metal hip arthroplasty revisions that they reported on).

b Updated report on Grammatopoulos et al. (2009)

### Defining ARMD

The term ARMD was introduced in 2010 as an umbrella term for painful MoMHA failures with one or more of the following features in the absence of another plausible diagnosis (Langton et al. 2010, Langton et al. 2011): large sterile effusions (including pseudotumor), macroscopic tissue necrosis, metallosis and ALVAL (or aseptic lymphocytic vasculitis associated lesions, which refers to a specific lymphocyte-dominated histopathological appearance). The term ARMD has generally been accepted as the most inclusive for describing this heterogeneous condition, which can have varying degrees of disease severity (discussed above in “Intra-operative findings and surgical strategy”). Continued research in this particular field has resulted in the definition for ARMD evolving with time. We would make a diagnosis of ARMD in MoMHAs if there was cross-sectional imaging and intra-operative evidence of a pseudotumor (a cystic, solid, or mixed mass communicating with the hip joint), or if there was no pseudotumor but abnormalities including pathological effusions, significant metallosis, synovitis, tissue damage and/or necrosis (Pandit et al. 2008, Langton et al. 2010, Langton et al. 2011, Hart et al. 2012, Nishii et al. 2012, Lainiala et al. 2015). Ideally this would be supported by appropriate histopathological findings, namely evidence of lymphocytic infiltrates (including ALVAL) and a phagocytic macrophage response to metal wear debris, with or without tissue necrosis (Willert et al. 2005, Campbell et al. 2010, Grammatopoulos et al. 2013). However, we recognize that surgery is planned based on preoperative investigations with any histopathological diagnosis of ARMD typically available only after revision has been performed. ARMD can also coexist with other abnormalities (such as loosening, osteolysis, instability) in the same way, for example, infection can coexist with other abnormalities (De Smet et al. 2011, Matharu et al. 2014b).

Of the 15 unique studies reviewed, which reported outcomes following ARMD revision, all provided some definition for ARMD. However, only 11 studies provided enough details about both the clinical and histopathological features to satisfy our working diagnosis of ARMD (Grammatopoulos et al. 2009, De Smet et al. 2011, Rajpura et al. 2011, Liddle et al. 2013, Matharu et al. 2014b, Munro et al. 2014, Norris et al. 2014, Pritchett 2014, Cip et al. 2015, Lainiala et al. 2015, Liow et al. 2016). No study formally stated how it dealt with multiple revision indications (e.g., by using a hierarchy to establish the primary diagnosis for revision). However, it was clear from the data presented that most studies had reported cases of ARMD alone, as well as ARMD coexisting with other abnormalities (such as loosening, osteolysis, instability). In addition to this variability between studies when diagnosing ARMD it is important to note that the diagnosis can be subjective even when the same criteria are used, with various surgeons potentially interpreting any abnormal features differently. Although reviews of the literature like the present one are inherently limited by the quality and reporting of the published studies reviewed, the variability in how ARMD has been diagnosed in the different studies has important implications when making comparisons between studies and must be considered when interpreting our review of the literature.

### Complications and re-revision surgery

The frequency of complications (up to 68%) and re-revision surgery (up to 38%) were variable amongst the short-term studies but generally high (Table 1). In the first 16 ARMD revisions performed at 1 center there was an increase in the frequency of complications (50% to 69%) and re-revision (38% to 44%) from the initial study at a mean of 3 years follow-up (Grammatopoulos et al. 2009) to the report at a median of 10 years (Matharu et al. 2017b). Some studies did report implant survival rates following ARMD revision, which were 88% at both 3 years (Liow et al. 2016) and 5 years (Matharu et al. 2014b), and 56% at 10 years (Matharu et al. 2017b). Recent data from the NJR for England and Wales reported that following 2,535 ARMD revision procedures in MoMHA patients the 5-year implant survival rate was 90% (Matharu et al. 2017a). This was similar to the 5-year implant survival reported in the NJR following all-cause non-MoMHA revision surgery (88%–89% depending on bearing surface and component fixation) (NJR 2016). Another recent study comparing outcomes following all-cause acetabular only revisions in MoMHAs with all-cause acetabular only revisions in metal-on-polyethylene THAs observed similar incidences of local complications, dislocation, infection, and re-revision between the groups at up to 2-year follow-up (Penrose et al. 2016).

Early observations suggested short-term outcomes following ARMD revision were poor, with half of patients sustaining major complications and over one-third requiring further operations (Grammatopoulos et al. 2009). Similar observations were reported in subsequent small cohorts reporting their initial experience following ARMD revision surgery (Rajpura et al. 2011, Munro et al. 2014). Furthermore the frequency of complications and re-revision following the first ARMD revision cases were higher compared with MoMHA revisions for non-ARMD indications (fracture, loosening, infection), and compared with matched patients undergoing primary conventional THA (Grammatopoulos et al. 2009). The poor outcomes following ARMD revision were thought to relate to the invasive and destructive nature of these lesions (Pandit et al. 2008, Grammatopoulos et al. 2009, Langton et al. 2010, Haddad et al. 2011). In addition to regular MoMHA patient surveillance these initial observations led regulatory authorities and orthopedic surgeons to widely recommend performing early revision in MoMHAs with ARMD (Grammatopoulos et al. 2009, De Smet et al. 2011, Haddad et al. 2011, MHRA 2012, FDA 2013). It was thought that this strategy would improve outcomes following ARMD revision. Therefore over time surgeons subsequently adopted a lower threshold for performing revision for ARMD.

There is evidence to suggest that outcomes following ARMD revision in MoMHAs may have improved with time. The reason for this is likely multifactorial and may include regular patient surveillance, lowering of the threshold for performing revision, increasing surgical experience with ARMD revisions (including employing different strategies to manage soft-tissue and bone damage, and reduce ARMD recurrence), and patients now undergoing revision at longer intervals from primary surgery rather than early after MoMHA.

One single surgeon series observed that the frequency of complications and re-revisions were significantly reduced in their latest 31 ARMD revision cases compared with their first 17 revisions (De Smet et al. 2011). In addition to lowering the threshold for performing ARMD revisions in the later cases the surgeon made numerous other changes to their practice including more intensive surveillance with routine blood metal ion sampling, revising both HRA components, implanting larger ceramic femoral head sizes, and postoperatively using anti-dislocation braces. Furthermore outcomes following ARMD revision were not significantly inferior compared with the 65 non-ARMD HRA revisions performed by the same surgeon (De Smet et al. 2011). Although this was contrary to earlier observations (Grammatopoulos et al. 2009), we acknowledge that when this initial cohort were reviewed at extended follow-up there was no longer a difference in the frequency of complications and re-revision following ARMD revisions compared with non-ARMD revisions (Matharu et al. 2017b).

2 of the largest and more recent studies (Table 1) have reported the lowest frequency of complications and re-revision (Pritchett 2014, Lainiala et al. 2015). 1 study was a single surgeon series of 90 revisions (Pritchett 2014), and the other was the largest published study involving 215 ARMD revisions performed by six surgeons (Lainiala et al. 2015). Therefore ARMD revisions performed in specialist centers by experienced and high-volume MoMHA revision surgeons, not surprisingly, seem to also lead to better outcomes, which has been postulated previously (De Smet et al. 2011, Liddle et al. 2013). Recent registry data suggest a weak effect between the time interval between primary and revision for ARMD and re-revision rates, with revisions performed early after MoMHA more likely to require re-revision (Matharu et al. 2017a). This is plausible given MoMHAs are scarcely implanted now, thus the time interval between primary and revision for patients with MoMHAs now developing ARMD is increasing. It is therefore suspected that those presenting with ARMD nowadays will tend to have more benign disease and hence better outcomes following revision compared with the aggressive and destructive lesions observed in MoMHAs which required early revision for ARMD (Grammatopoulos et al. 2009, Munro et al. 2014).

A recent NJR analysis of MoMHA revisions compared the subsequent outcomes in 1,288 ARMD revisions matched to 1,288 non-ARMD revision indications (Matharu et al. 2018b). That study observed that patients revised for ARMD had approximately half the risk of re-revision compared with patients undergoing non-ARMD revision surgery. This was contrary to the initial observations (Grammatopoulos et al. 2009, Rajpura et al. 2011, Munro et al. 2014). The authors concluded that surgeons and worldwide regulatory authorities have positively influenced outcomes following ARMD revision by promoting regular patient surveillance and widely recommending a lower threshold for performing revision surgery (Matharu et al. 2018b). Interestingly the highest risk of re-revision surgery was when MoMHAs were revised for infection and dislocation/subluxation (5-year implant survival rates of 81% and 82% respectively) (Matharu et al. 2018b).

### Reasons for re-revision surgery

In line with previous evidence (Matharu et al. 2014a), the commonest indications for re-revision surgery following ARMD revision in the included studies were dislocation, ARMD recurrence, aseptic component loosening, and infection (Table 1).

Dislocation can be related to ARMD lesions that are destructive and/or require extensive soft-tissue debridement. Both can compromise the hip abductors and/or short external rotators and therefore lead to hip instability (De Smet et al. 2011). Other risk factors for dislocation include reducing the large-diameter MoM bearing when exchanging to a non-MoM articulation, and not correcting any residual component malposition at ARMD revision (for example when performing modular revisions in MoM THAs) (Stryker et al. 2015). Using large femoral head sizes (>36 mm) at ARMD revision has not prevented dislocations (Munro et al. 2014). This suggests that instability following ARMD revision is a complex problem, with some surgeons using anti-dislocation braces, dual-mobility acetabular components, and an overall lower threshold for performing ARMD revision procedures in an attempt to reduce the subsequent dislocation risk (De Smet et al. 2011, Pritchett 2014).

Most studies have reported at least 1 case of ARMD recurrence following ARMD revision (see Table 1). Recurrence can occur due to incomplete excision of metal debris at revision. Reasons for incomplete excision include surgical error and being unable to excise all the metal debris because of its proximity to vital neurovascular structures (Grammatopoulos et al. 2009, Liddle et al. 2013). In such cases serial post-revision cross-sectional imaging can be useful for monitoring progression of any residual ARMD (Grammatopoulos et al. 2009, Munro et al. 2014). Recurrence of ARMD can also occur if there is another potential source of metal wear debris and/or corrosion, including implantation of another MoM bearing (Liddle et al. 2013, Matharu et al. 2014b, Pritchett 2014) or using a cobalt-chrome femoral head on a titanium alloy or cobalt-chrome femoral stem (Munro et al. 2014, Whitehouse et al. 2015, Plummer et al. 2016). In light of these observations most surgeons now use ceramic femoral heads when performing ARMD revisions (Grammatopoulos et al. 2009, De Smet et al. 2011, Liddle et al. 2013), with a taper adapter used if the stemmed femoral component is retained given the theoretical risk of ceramic head fracture when impacted onto retained tapers (Plummer et al. 2016).

Aseptic loosening, usually of the acetabular component, may be due to invasive and destructive ARMD causing significant osteolysis and/or iatrogenic bone loss when removing a well-fixed component, which increases the risk of failed implant osseointegration. 1 study experienced a number of failures due to aseptic acetabular loosening despite the absence of bone defects, but this was thought to be due to the fiber metal-backed acetabular components implanted at revision, with better results obtained when porous tantalum components were used (Munro et al. 2014).

Risk factors for infection may include multiple operations, incomplete excision of metal debris or necrotic tissue at revision, and the retention of primary MoMHA components (Liddle et al. 2013, Munro et al. 2014).

### Functional outcomes

Patient-reported outcomes after the initial ARMD revisions were poor (Grammatopoulos et al. 2009, Ebreo et al. 2011, Munro et al. 2014), with these also being inferior compared with MoMHA revisions for non-ARMD indications, and compared with primary conventional THAs (Grammatopoulos et al. 2009). The poor functional outcomes reported in an early study of 16 ARMD revisions (Grammatopoulos et al. 2009) persisted at extended follow-up, and remained inferior compared with MoMHA revisions for non-ARMD indications (Matharu et al. 2017b). These poor functional outcomes were also considered a consequence of the invasive and destructive nature of ARMD lesions, with a lower threshold for performing such revision procedures widely adopted (Grammatopoulos et al. 2009, De Smet et al. 2011, Haddad et al. 2011, MHRA 2012, FDA 2013).

The majority of subsequent studies have reported good or excellent functional outcomes following ARMD revision (De Smet et al. 2011, Rajpura et al. 2011, Liddle et al. 2013, Su and Su 2013, Matharu et al. 2014b, Pritchett 2014, Cip et al. 2015, Lainiala et al. 2015), defined as a mean Oxford Hip Score (OHS) of 34 or above or a Harris Hip Score (HHS) of 80 or above (Harris 1969, Kalairajah et al. 2005, Murray et al. 2007). However, not all of the more recent studies achieved good functional outcomes following ARMD revision (Norris et al. 2014, van Lingen et al. 2015, Liow et al. 2016), which suggests other factors may influence outcomes after these procedures.

### Predicting re-revision and guiding surgical decisions

Identifying prognostic factors of outcome following ARMD revision would provide surgeons with thresholds for performing revision and help guide decisions regarding the type of reconstruction to perform. However, most of the evidence regarding factors predictive of poor outcomes following ARMD revision surgery is limited to small studies that were not adequately powered to identify prognostic factors (De Smet et al. 2011, Liddle et al. 2013, Matharu et al. 2014b, Liow et al. 2016).

In some of the earliest MoMHAs revised for ARMD a significantly higher risk of re-revision and inferior functional outcomes were reported when another MoM bearing was implanted at revision (Liddle et al. 2013, Matharu et al. 2014b, Pritchett 2014). This is suspected to be due to the continued source of metal wear debris and/or corrosion, and has led to using non-MoM bearing surfaces at ARMD revision. Solid pseudotumors have been a risk factor for re-revision surgery following ARMD revision (Liddle et al. 2013). Similarly a recent study of 102 ARMD revisions identified MARS-MRI evidence of solid lesions with abductor deficiency to be predictive of post-revision complications, with pre-revision radiographic implant loosening also predicting poor outcomes (Liow et al. 2016). However, this study did not find other factors to be predictive of post-revision complications, such as pre-revision blood metal ions, type of revision articulation, and femoral head size (Liow et al. 2016).

Recent analysis of NJR data identified 4 predictors of re-revision surgery following ARMD revision in MoMHAs: high BMI at revision, modular component only THA revisions (exchange of the head and liner only with/without taper adapter), ceramic-on-ceramic revision bearings, and acetabular bone grafting (Matharu et al. 2017a). The authors concluded that as these predictors included modifiable factors (type of THA revision procedure, revision bearing surface, and possibly the use of acetabular bone graft) surgeons could potentially reduce failure rates further following ARMD revision. By contrast, smaller studies from the Australian Joint Replacement Registry have reported that re-revision following aseptic MoM HRA revision surgery was not influenced by the type of revision procedure performed or the revision bearing surface implanted (de Steiger et al. 2010, Wong et al. 2015).

## Conclusions

Numerous studies have now reported on the short- to medium-term risks associated with ARMD revision surgery in MoMHA patients, which can be used to counsel patients informatively pre-revision. Evidence suggests that outcomes following ARMD revision may have improved with time. The reason for this is multifactorial and may include regular patient surveillance, lowering of the threshold for performing revision, surgical experience with ARMD revisions, and patients now undergoing revision at longer intervals from primary surgery with such cases potentially being less severe compared with revisions performed early after MoMHA. However, we consider that the threshold for performing ARMD revision surgery need not be lowered much further as this introduces the potential for surgical risk to outweigh any benefits.

By contrast, robust thresholds for performing ARMD revision surgery are lacking. Although non-registry studies have attempted to identify predictors of cross-sectional imaging progression (Reito et al. 2014, Briant-Evans et al. 2015, Kwon et al. 2016, Matharu et al. 2016a), and predictors of poor outcomes following ARMD revision (De Smet et al. 2011, Liddle et al. 2013, Matharu et al. 2014b, Dimitriou et al. 2016), these have been short-term studies that were underpowered for identifying predictors. Recent registry data suggest that modifiable factors exist which may allow surgeons to reduce failure rates further following ARMD revision (Matharu et al. 2017a), although these require validation.

Future studies should focus on obtaining good quality evidence to develop robust thresholds for performing ARMD revision surgery and to inform the surgical strategy at ARMD revision, which will both assist surgeons when managing MoMHA patients. Most of these questions require large well-designed multi-center studies with extended follow-up that can assess potentially important predictors of complications, re-revision, and functional outcomes following ARMD revision surgery (such as pre-revision blood metal ion levels and cross-sectional imaging findings). These issues simply cannot be addressed using current arthroplasty registries, which do not collect such data. Multi-center studies would provide important information on the role of blood metal ions and cross-sectional imaging in predicting disease severity, tissue destruction, and outcomes following ARMD revision. This information can then be used to guide thresholds for performing ARMD revision. Both multi-center studies and retrospective registry cohorts are required to inform the surgical strategy at ARMD revision, which will include establishing the optimal implant fixation methods, the optimal bearing surface (comparing highly cross-linked polyethylene articulating with ceramic vs. metal heads), and the management of instability in association with soft-tissue problems (dual mobility cups vs. constrained acetabular liners). Finally, the long-term systemic effects of metal ion exposure in MoMHA patients must also be investigated, particularly the potential oncological and cardiac consequences. This will also help establish whether there are any systemic reasons to recommend revision of MoMHAs in the absence of local ARMD. Addressing the outlined research questions would allow a more evidence-based approach for the management of MoMHA patients who may require revision for ARMD.

### Supplementary data

The Appendix is available in the online version of this article, http://dx.doi.org/10.1080/17453674.2018.1440455.

GSM: literature review, manuscript draft, and revision. AJ, AE, HGP, DWM: contributed to the literature review, and manuscript revision.

The authors would like to thank the following organizations for funding this research: Arthritis Research UK (grant reference number 21006), the Royal College of Surgeons of England, the Orthopaedics Trust and the Royal Orthopaedic Hospital Hip Research and Education Charitable Fund.

GM has undertaken medico-legal work for Leigh Day, which includes work relating to metal-on-metal hip replacements. AJ has undertaken medico-legal work for Freshfields Bruckhaus Deringer, which includes work relating to metal-on-metal hip replacements. HGP provides expert testimony to Kennedys Law, which includes work relating to metal-on-metal hip replacements. None of the other authors have any conflicts of interest relating specifically to this work. No commercial companies were involved in the planning of this review, analysis and interpretation of data, or writing of the manuscript. DWM has undertaken medico-legal work for Herbert Smith Freehills, which includes work relating to metal-on-metal hip replacements

*Acta* thanks Johan Kärrholm and other anonymous reviewers for help with peer review of this study.

## Supplementary Material

IORT_A_1440455_SUPP.PDF
