# Supplementary material for: Revision surgery of metal-on-metal hip arthroplasties for adverse reactions to metal debris: A clinical update
Source: Acta Orthop. 2018 Mar 1;89(3):278–88. doi: 10.1080/17453674.2018.1440455 (PMC6055775; doi:10.1080/17453674.2018.1440455)
Supplement: IORT_A_1440455_SUPP.PDF [file IORT_A_1440455_SM5229.pdf]

Supplementary data

Appendix

A systematic review undertaken in 2013 identified six studies reporting the outcomes following 216 MoMHA revisions performed for ARMD (Matharu et al. 2014a). This systematic review was updated in December 2016 using the same methods described in the initial paper (summarized below) to identify any additional relevant publications reporting the outcomes of more than 10 HRA and/or more than 10 MoM THA revision procedures performed for ARMD. This criterion was used to eliminate very small case series that may have been influenced by learning curves, and to minimize any selection and publication bias.

The search was performed independently by 2 authors (GSM and HGP) using PubMed, Medline, Embase, and the Cochrane Central Register of Controlled Trials to identify relevant articles published between January 1, 2009 and December 31, 2016. Combinations of the following search terms were used in each electronic databases: "adverse reaction to metal debris (ARMD)," "adverse local tissue reaction (ALTR)," "aseptic lymphocytic vasculitis-associated-lesions (ALVAL)," "pseudotumour," "pseudotumor," "metal-on-metal," "hip resurfacing," "total hip replacement," "total hip arthroplasty," "failure," "revision surgery," "outcomes," "clinical outcomes," and "functional outcomes". No restrictions were placed on language, study type, or publication status. Full-text articles were obtained of any potentially relevant studies. All articles obtained were reviewed independently by both of the authors. Additional review was also performed of pertinent references from the bibliographies of these publications not found in the electronic search but fitting the inclusion criteria. There were no restrictions placed on the minimum follow-up period of patients following revision in the relevant studies identified. Any duplicate publications identified from searching the 4 electronic databases were also excluded.

The methodological quality of each included study was assessed using the Oxford Centre for evidence-based medicine levels of evidence. All assessments were independently undertaken by two authors with any disagreement resolved by discussion and consensus with the third author (DWM).

All study data were independently extracted and recorded in relevant data tables by two authors. Data extracted included: patient demographics (age and gender), implants requiring revision for ARMD, time from index arthroplasty to ARMD revision, follow-up time, and clinical outcomes after ARMD revision surgery. Outcomes of interest following ARMD revision surgery were the frequency of all complications (intraoperative and post-revision), the frequency of re-revision surgery, and post-revision functional outcomes. If studies reported on functional outcome following revision hip arthroplasty, the specific instrument used and method of scoring was extracted from the original report and recorded to assist comparison between studies. The mean and range of time patients were followed up in each study after revision surgery was also documented to assist with interpretation of the clinical outcomes of interest. Meta-analysis of the pooled data was not performed due to the heterogeneity between studies in terms of implants used and data reported.
